# Comparative Genomics of Closely Related *Tetragenococcus halophilus* Strains Elucidate the Diversity and Microevolution of CRISPR Elements

**DOI:** 10.3389/fmicb.2021.687985

**Published:** 2021-06-18

**Authors:** Minenosuke Matsutani, Takura Wakinaka, Jun Watanabe, Masafumi Tokuoka, Akihiro Ohnishi

**Affiliations:** ^1^NODAI Genome Research Center, Tokyo University of Agriculture, Tokyo, Japan; ^2^Manufacturing Division, Yamasa Corporation, Choshi, Japan; ^3^Department of Fermentation Science, Faculty of Applied Bio-Science, Tokyo University of Agriculture, Tokyo, Japan

**Keywords:** *Tetragenococcus halophilus*, CRISPR elements, bacteriophage-resistance, comparative genomics, microevolution

## Abstract

*Tetragenococcus halophilus* – a halophilic lactic acid bacterium – is frequently used as a starter culture for manufacturing fermented foods. *Tetragenococcus* is sometimes infected with bacteriophages during fermentation for soy sauce production; however, bacteriophage infection in starter bacteria is one of the major causes of fermentation failure. Here, we obtained whole-genome sequences of the four *T. halophilus* strains YA5, YA163, YG2, and WJ7 and compared them with 18 previously reported genomes. We elucidated five types of clustered regularly interspaced short palindromic repeat (CRISPR) loci in seven genomes using comparative genomics with a particular focus on CRISPR elements. CRISPR1 was conserved in the four closely related strains 11, YA5, YA163, and YG2, and the spacer sequences were partially retained in each strain, suggesting that partial deletions and accumulation of spacer sequences had occurred independently after divergence of each strain. The host range for typical bacteriophages is narrow and strain-specific thus these accumulation/deletion events may be responsible for differences in resistance to bacteriophages between bacterial strains. Three CRISPR elements, CRISPR1 in strains 11, YA5, YA163, and YG2, CRISPR2 in strain WJ7, and CRISPR2 in strain MJ4, were inserted in almost the same genomic regions, indicating that several independent insertions had occurred in this region. As these elements belong to class 1 type I-C CRISPR group, the results suggested that this site is a hotspot for class 1, type I-C CRISPR loci insertion. Thus, *T. halophilus* genomes may have acquired strain-specific bacteriophage-resistance through repeated insertion of CRISPR loci and accumulation/deletion events of their spacer sequences.

## Introduction

*Tetragenococcus halophilus* is a halophilic lactic acid bacterium that is abundant in various salted foods such as soy sauce, salted fish, and vegetable pickles (Chen et al., 2006; Satomi et al., 2008; Tanaka et al., 2012). During fermentation of these products, *T. halophilus* plays an important role in the production of organic acids, amino acids, and flavoring compounds (Udomsil et al., 2010, 2017; Lee et al., 2018). In traditional breweries, microorganisms that survived the fermentation process were repeatedly used as starter cultures for subsequent fermentation batches. Currently, however, selected strains of *T. halophilus* are frequently used as fermentation starters to prevent biogenic amine accumulation in the products (Kuda et al., 2012; Wakinaka et al., 2019).

The food fermentation industry relies on selected bacterial strains as starter cultures; however, bacteriophage infection is a cause of fermentation failure (Leroy and De Vuyst, 2004). Bacteriophages infecting *T. halophilus* have been isolated from fermenting soy sauce mash (Uchida and Kanbe, 1993; Higuchi et al., 1999), and their host range is narrow and strain specific. Currently isolated bacterial strains may have survived for several generations in the presence of bacteriophages; however, anti-phage mechanisms that determine phage susceptibility of this species remain unknown. The clustered regularly interspaced short palindromic repeat (CRISPR)/CRISPR-associated (Cas) system is a bacterial defense system preventing bacteriophage infection (Deveau et al., 2010; Garneau et al., 2010). CRISPR arrays consist of short repeats separated by unique spacers derived from foreign nucleic acids. These spacers are transcribed to RNAs that elicit immune responses counteracting invading nucleic acids, including bacteriophage genomes. In most cases, *cas* genes responsible for immune functions occur adjacent to the CRISPR array.

Here, we report the draft genome sequences of the four *T. halophilus* strains YA5, YA163, YG2, and WJ7; the three former strains were isolated from soy sauce mash, and the latter was isolated from picked fish, termed *nukazuke* (Wakinaka and Watanabe, 2019; Shirakawa et al., 2020). We also compared CRISPR loci of different *T. halophilus* strains. As the host range of typical bacteriophages is narrow and strain specific, we also examined CRISPR elements in other bacterial strains of the same genetic lineage (Uchida and Kanbe, 1993; Higuchi et al., 1999; Spus et al., 2015).

## Materials and Methods

### Bacterial Strains and Culture Conditions

*Tetragenococcus halophilus* of the four strains, YA5, YA163, YG2, and WJ7, were cultured in De Man, Rogosa, Sharpe medium (Becton Dickinson, Franklin Lakes, NJ, United States) supplemented with 10% NaCl, at 30°C, and under static conditions.

### Genomic DNA and Library Preparation and Genome Sequencing, Assembly, and Annotation

Genomic DNA of strains YA5, YA163, YG2, and WJ7 was isolated using the DNeasy PowerSoil Pro Kit (QIAGEN Sciences, Germantown, MD, United States) and the automated QIAcube system (QIAGEN Sciences). Quantity and purity of genomic DNA were assessed using a Qubit 2.0 Fluorometer with a Qubit dsDNA BR Assay Kit (Thermo Fisher Scientific, Inc., Waltham, MA, United States) and a NanoDrop 1000 spectrophotometer (Thermo Fisher Scientific). Genomic DNA library was prepared using the Illumina Nextera DNA Flex Library Prep Kit (Illumina, San Diego, CA, United States) according to the manufacturer’s instructions. Whole genome sequencing was performed using paired-end sequencing strategy (2 × 300 bp) on an Illumina MiSeq sequencing platform (Illumina). Adapter sequences and low-quality regions were trimmed using Trim Galore! v.0.6.4 with default settings^1^. A *de novo* assembly of trimmed genome sequences was performed using SPAdes v. 3.13.0 (Bankevich et al., 2012). The resulting contigs were aligned against the complete genome sequence of *T. halophilus* subsp. *flandriensis* strain LMG 26042^*T*^ (RefSeq assembly accession: GCF_003795105.1) using Mauve v.2.3.1 (Darling et al., 2004, 2010). Gene detection and genome annotation of the draft genome assemblies were performed using the DDBJ Fast Annotation and Submission Tool with default settings (Tanizawa et al., 2016, 2018). The resulting assemblies were used for comparative genome analysis. Genome sequences of 18 *T. halophilus* strains were downloaded from the NCBI Reference Sequence Database (RefSeq) (Supplementary Table 1; Tatusova et al., 2014).

### Average Nucleotide Identity Based on MUMmer Calculation and Heatmapping of ANIm Matrix

The average nucleotide identity based on MUMmer (ANIm) was calculated using the MUMmer 4.0.0 beta2 package (Goris et al., 2007; Richter and Rosselló-Móra, 2009; Marçais et al., 2018). ANIm values were generated based on the NUCmer alignment for pairwise comparisons of the 22 closely related genomes. A heatmap of ANIm matrix was constructed using the average_nucleotide_identity.py script included in the Pyani package with the “-m ANIm –g” option (Pritchard et al., 2016).

### Detection and Graphical Representation of CRISPR Element Gene Clusters

Candidate CRISPR elements in the 22 genomes were extracted using MinCED 0.3.0 with default settings (Bland et al., 2007). Contigs detected as candidate CRISPR elements were further examined using recently reported tools. The *cas* genes and orientation of CRISPR arrays from genome fragments comprising candidate CRISPR elements were also investigated with CRISPRidentify package (Mitrofanov et al., 2021). CRISPR arrays with certainty scores ≥0.75 were considered true CRISPR arrays. The *cas* genes were also predicted using the CRISPRCasFinder server (Couvin et al., 2018). Figures for comparing CRISPR element gene clusters were produced using genoPlotR 0.8.9 (Guy et al., 2010). To identify amino acid sequence identities among homologous proteins, a BLASTP search was performed with a sequence overlap (query and subject) ≥50% (Altschul et al., 1997).

### Whole Genome Alignment and Genome Map Construction

Genome sequences of the seven *T. halophilus* strains 11, YA5, YA163, YG2, WJ7, MJ4, and KUD23 were used as queries for the whole genome alignment against the genome sequence of *T. halophilus* NBRC 12172. These genomes were independently aligned against that of strain NBRC 12172 using a NUCmer (Marçais et al., 2018). CRISPR insertion points of each genome were identified from NUCmer alignment, and the adjacent 10,000 bp sequences of reference genome data were extracted and mapped to reference sequences using NUCmer within the MUMmer 4.0.0 beta2 package with default option (Marçais et al., 2018). We produced graphic illustrations of genome alignments using CGView (Stothard and Wishart, 2005).

### Construction of a Genome-Based Phylogenetic Tree

For genome-based phylogenetic analysis, we retrieved orthologous gene sets from the target data set using a reciprocal best-hits search with a BLASTP E-value cutoff of 10^–10^ and sequence overlap (query and subject) ≥70%. Each orthologous gene set was aligned using MSAProbs v0.9.7 at the amino acid level and was back-translated into nucleotide sequences (Liu et al., 2010; Liu and Schmidt, 2014). Poorly aligned regions were removed using GBLOCKS 0.91b (Talavera and Castresana, 2007). A phylogenetic tree for each orthologous gene set was constructed using the GTRGAMMA model in RAxML 8.2.2 (Stamatakis, 2006; Stamatakis, 2014). Alignments of all genes were concatenated, and a tree search was performed using the GTRGAMMA model in RAxML 8.2.2. A phylogenetic tree was drawn using the MEGA X package (Kumar et al., 2018).

## Results and Discussion

### Genome Features of Four Newly Sequenced Draft Genomes

We obtained draft genome sequences of the four *T. halophilus* strains, YA5, YA163, YG2, and WJ7 using an Illumina MiSeq sequencing platform (Illumina), which generated 1,686,583, 1,923,062, 1,702,184, and 1,952,698 paired-end reads, respectively. *De novo* assembly generated high-quality contigs for comparative genomic analyses. The total genome size of these strains ranged from 2,318,625 to 2,443,531 bp with 35.56–35.72% average G + C content. The estimated sequence coverage of the genomes of strains YA5, YA163, YG2, and WJ7 was 414−, 477−, 440−, and 494-fold, respectively. The genome characteristics of the four strains are summarized in Table 1. The number of contigs ranged from 113 to 132, and their N_50_ values ranged from 41,027 to 44,198 bp. There were 2,400, 2,375, 2,254, and 2,317 protein-coding genes in the genomes of strains YA5, YA163, YG2, and WJ7, respectively. Moreover, 3 rRNA genes, 1 tmRNA gene, and 53–55 tRNA genes were predicted from the four assembled genomes (Table 1). Thus, the genome characteristics of the four strains were similar, suggesting that these strains were closely related. Of the eighteen previously reported genomes of *T. halophilus*, only three strains retained CRISPR elements in their genomes; however, genomes of all four strains assembled in this study possessed CRISPR elements. There were two, one, two, and one CRISPR elements in the genomes of strains YA5, YA163, YG2, and WJ7, respectively.

**TABLE 1:** Characteristics of whole genome sequences of the four *Tetragenococcus halophilus* strains assembled in this study.

Strain name	YA5	YA163	YG2	WJ7
Assembly size (bp)	2,318,625	2,420,090	2,372,244	2,443,531
Total number of contigs	122	132	117	113
GC content (%)	35.72	35.70	35.67	35.56
Longest contig (bp)	119,288	125,671	158,547	213,609
N_50_ (bp)	41,027	43,881	44,018	44,198
L_50_	18	19	17	16
Estimated fold coverage	440	477	494	414
CDS	2,254	2,375	2,317	2,400
rRNA genes	3	3	3	3
tRNA genes	53	54	54	55
tmRNA genes	1	1	1	1
DRA accession no.	DRR220996	DRR220995	DRR220997	DRR220994
GenBank accession no.	BLRO01000001-BLRO01000122	BLRN01000001-BLRN01000132	BLRP01000001-BLRP01000117	BLRM01000001-BLRM01000113

### Average Nucleotide Identity Based on MUMmer Clustering of *T. halophilus* Genomes

**FIGURE 1:**
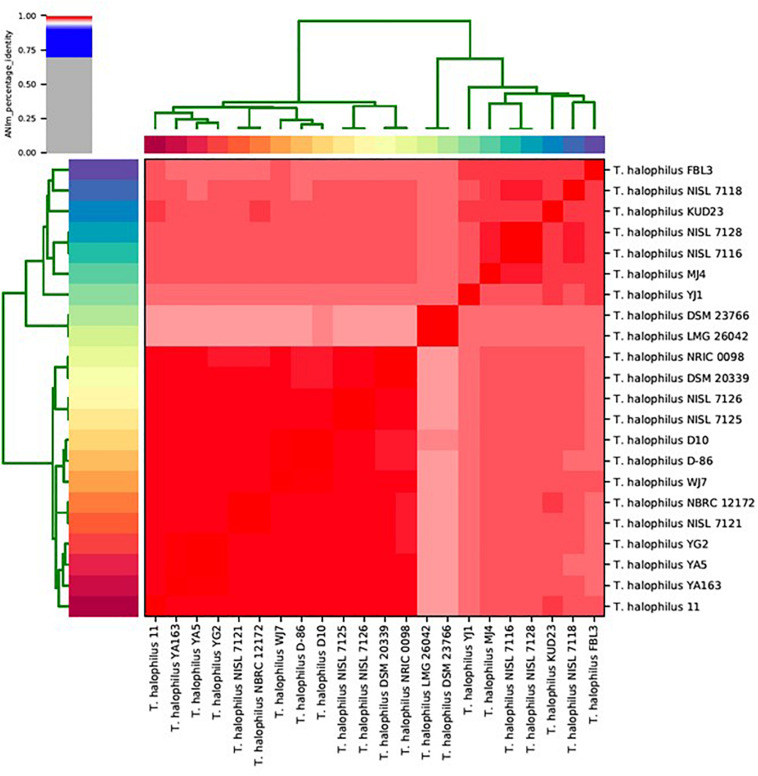
Matrix and clustering of average nucleotide identity based on MUMmer (ANIm) identification of 22 *Tetragenococcus* strains. Red color in the heatmap indicate highly conserved genome pairs.

We calculated ANIm against all-to-all genomes for *T. halophilus* (Goris et al., 2007; Richter and Rosselló-Móra, 2009). ANIm clustering showed that the *T. halophilus* strains were divided into two large sub-groups (Figure 1). The type strains of *T. halophilus* subsp. *halophilus*, strains DSM 20339^T^ and NRIC 0098^T^, were clustered into the largest clade comprising 13 genomes. Therefore, we considered this group to be a clade of *T. halophilus* subsp. *halophilus*. Since the genomes of the four strains from this study were also clustered into this clade, we considered these strains to be typical *T. halophilus* subsp. *halophilus* strains. Of these, strain WJ7 was most closely related to strains D-86 and D10. Strains YA163, YA5, and YG2 were closely related to each other, and their sub-clade comprised strain 11 along with the three strains YA163, YA5, and YG2 (Figure 1). Two strains, LMG 26042^T^ and DSM 23766^T^, which are type strains of *T. halophilus* subsp. *flandriensis*, clustered in a different clade (Justé et al., 2012).

### Comparison of CRISPR Elements

Clustered regularly interspaced short palindromic repeat elements and their accumulated spacer sequences play crucial roles in conferring resistance to bacteria against bacteriophages (Barrangou et al., 2007). Bacteriophage-resistance is an important trait of a bacterial strain that is used as a starter strain for production of fermented food. Therefore, we performed comparative genomics with a particular focus on CRISPR elements in *Tetragenococcu*s genomes. To investigate the distribution of CRISPR elements in the 22 genomes, we extracted repeat-spacer arrays of CRISPR elements and CRISPR-related genes (Bland et al., 2007; Mitrofanov et al., 2021). As a result, ten CRISPR elements were identified in the genomes of the seven strains 11, YA163, YA5, YG2, WJ7, MJ4, and KUD23. All CRISPR elements with strand and certainty score are shown in Supplementary Table 2. CRISPR arrays with certainty scores ≥0.75 were considered true CRISPR arrays in the CRISPRidentify package (Mitrofanov et al., 2021). The *cas* genes were predicted with CRISPRidentify package and CRISPRCasFinder server, and results produced using the CRISPRidentify package are summarized in Supplementary Table 3 (Mitrofanov et al., 2021). All spacer sequences are shown in Supplementary Table 4. Strains YA5, YG2, WJ7, and MJ4 possessed more than two sets of CRISPR elements. Based on gene organization and repeat and spacer sequences, we classified the ten CRISPR elements into five groups, CRISPR1 to 5 (Supplementary Table 2). Of these, CRISPR3 showed certainty scores ≤0.75. Therefore, we considered CRISPR3 a possible candidate. Although the Cas proteins in groups CRISPR4 and 5 showed amino acid identity ≥90%, their spacer sequences showed no overlap; therefore, they were divided into two groups. Makarova et al. reported a new evolutionary classification of CRISPR-Cas systems and *cas* genes (Makarova et al., 2020). Based on their classification, all CRISPR elements detected in *T. halophilus* genomes are classified as class 1 CRISPR-Cas systems, and this class has many subtypes. Hence, groups CRISPR1 to 5 were further divided into subtypes I-A, I-A, I-D, III-A, and III-A, respectively. It was observed that the same subtypes had the same repeat sequences in their repeat-spacer array. The repeat sequences of subtypes I-A, I-D, and III-A were “GTCGCTCTCTTCGTGAGAGCGTGGATTGAAAT,” “GTCT TTCCCGCATAAGCGGGGGTGATCC,” and “AATAGATAC CTAACCCCATTATTAGGGGACGAGAAC,” respectively, although some mutations occurred in some of the repeat sequences.

**FIGURE 2:**
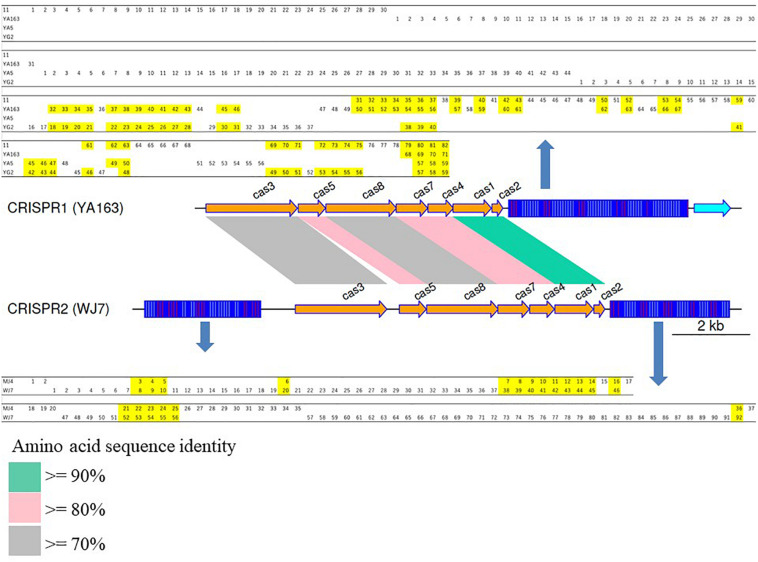
Graphical representation of the CRISPR gene clusters for CRISPR1 (strain YA163) and CRISPR2 (strain WJ7). The figure was produced using genoPlotR 0.8.9 (Guy et al., 2010). Spacer sequences completely conserved in more than two strains are highlighted with yellow. Spacer numbers used in this figure are also listed in Supplementary Table 4.

Since groups CRISPR1 to 3 had more than two members each, a comparison of the spacer sequences conserved in the same group was performed. Graphical representation and comparison data are shown in Figure 2 and Supplementary Table 5, respectively. Although groups CRISPR1 and 2 had the same repeat pattern and gene organization, their spacer sequence and amino acid sequence identities implied that the groups were different. The CRISPR1 sequence was conserved in the four closely related strains 11, YA163, YA5, and YG2, which revealed that the spacer sequences were partially retained in each of the strains, and the 3′-end of each element retained several common spacer sequences with partial deletion. Garrett et al. reviewed some aspects concerning Spacer Dynamics in the CRISPR Array. The terminal spacer-repeat unit rarely participates in rearrangements, possibly because of polymorphisms, and the last spacer-repeat unit is stable (Garrett, 2021). As shown in Figure 2, all CRISPR elements belonging to group CRISPR1 also possess an array identical to that of the last spacer-repeat unit. Meanwhile, the 5′-end of each element had only unique spacer sequences. This suggests that the repeated partial deletions of spacer sequences from the repeat-spacer array occurred independently after the divergence of each strain with accumulation of new spacer sequences. Group CRISPR3 also showed the same trend. Strains YA5 and YG2 possessed 3 and 15 unique spacer sequences at the 5′-end of the CRISPR elements, respectively (see Supplementary Table 5). Barrangou et al. (2007) reported that the spacer sequences are directly related to bacteriophage-resistance. Thus, these variations may directly contribute to strain-specific bacterial resistance.

### Comparison of CRISPR Elements Insertion Site

**FIGURE 3:**
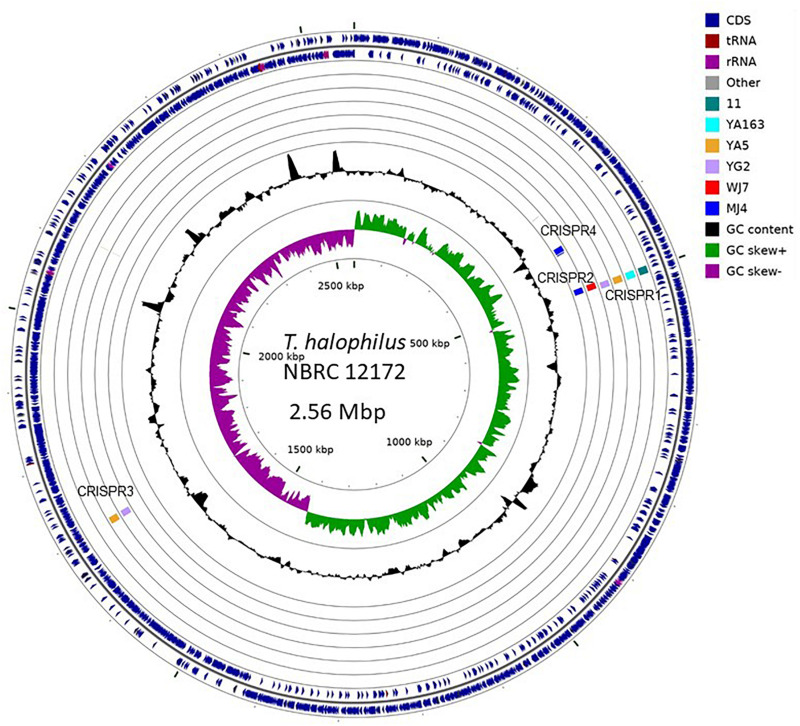
Graphical representation of CRISPR inserted regions. Insertion points were identified with NUCmer alignment (Marçais et al., 2018). Each query genome was independently aligned against the complete genome sequence (GenBank acc. No.: AP012046.1) of the *Tetragenococcus halophilus* NBRC 12172 strain. The illustration was produced using CG-view (Stothard and Wishart, 2005).

To investigate genomic regions containing CRISPR elements, we performed whole genome alignment against the genome of *T. halophilus* NBRC 12172, which has no CRISPR element inserted-regions, using the genome sequences of seven strains, 11, YA5, YA163, YG2, WJ7, MJ4, and KUD23, as a query (Marçais et al., 2018). We could not assign the inserted region of CRISPR5 to the genome of strain NBRC 12172. The surrounding 10 kb regions of CRISPR insertion points of nine CRISPR elements excluding CRISPR5 from strain KUD23 are shown in Figure 3. As a result, CRISPR elements belonging to the same group were observed to occur in the same genomic regions.

**FIGURE 4:**
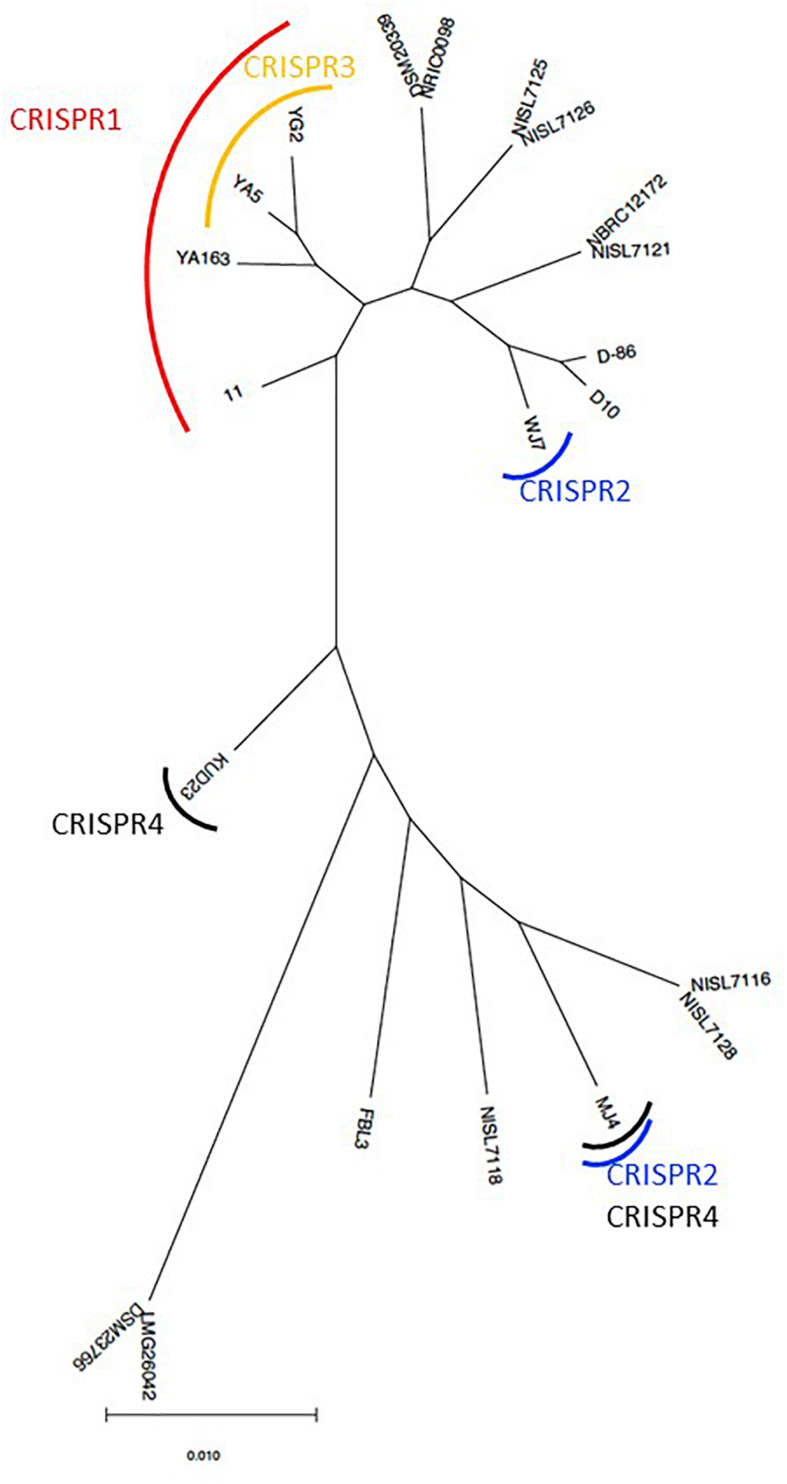
Maximum-likelihood phylogenetic tree of 22 *Tetragenococcus halophilus* strains. The tree was prepared based on the nucleotide sequences from 1,292 orthologous gene sets. The strains that retained CRISPR loci in the genome sequence are indicated by a curved line.

Based on the phylogenetic relationship of *T. halophilus* genomes, the distribution of CRISPR loci was investigated (Figure 4). CRISPR1 was conserved in four strains: 11, YA163, YA5, and YG2. As described above, these strains were closely related (Figures 1, 4). This indicates that the common ancestor of these strains acquired these CRISPR elements before the divergence of each strain. CRISPR3 element was conserved in strains YA5 and YG2, and these two strains were the most closely related. This also indicated that the common ancestor of strains YA5 and YG2 acquired this CRISPR element. However, the CRISPR2 sequence occurred in strains WJ7 and MJ4. As these strains were phylogenetically distinct from each other, the CRISPR2 element may have been independently inserted into their genomes (Figure 4). Three CRISPR elements, CRISPR1 in four strains, CRISPR2 in WJ7, and CRISPR2 in MJ4, were inserted in almost the same genomic regions, indicating that two or three independent insertions occurred in this region (Figure 3). Since these elements belong to class 1, type I-C CRISPR group, the results suggest that this site is a hotspot for class 1, type I-C CRISPR loci insertion.

Peters et al. (2017) reported that some minimal class 1, type I-F CRISPR-Cas systems, and truncated type I-B CRISPR-Cas systems were inserted through *Tn7*-like transposons. Recently, type I-F, including Tn7-like transposons, were re-classified as class 1, type I-F3 CRISPR elements (Makarova et al., 2020). However, the insertion pathways of class 1, type I-C CRISPR loci remains unknown. Although, we compared the gene repertoire of transposons surrounding type I-C CRISPR elements, we could not identify any Tn7-like transposons. CRISPR2 element possessed *IS110* family and IS*Lre2* family insertion sequences. Of these, IS*Lre2* was conserved only in the genome of strain WJ7 (data not shown). In contrast, CRISPR1 element in the four strains sequenced in this study showed no transposase in its surrounding regions. This suggests a different insertion pathway of type I-C CRISPR elements. Comparison of two phylogenetically distinct genomes, LMG 26042 and NBRC 12172, which do not possess type I-C CRISPR elements indicated that the dihydroxyacetone kinase operon *dhaMKL* was conserved in the CRISPR insertion site (data not shown). As this operon was eliminated from all type I-C CRISPR loci inserted genomes, there may be a signal region surrounding this operon for insertion of class 1, type I-C CRISPR loci.

Comparative genomic studies on these four strains and previously reported 18 *T. halophilus* strains showed significant variations in the CRISPR arrays at the genome level. Their accumulated spacer sequences indicate previous encounters with bacteriophages. The distinct CRISPR arrays at almost the same chromosomal location suggest a hotspot for the insertion of CRISPR loci.

## Conclusion

We revealed insertion points of nine CRISPR elements in the genome of *T. halophilus*. Especially high-frequency insertions occurred in hotspots located at specific chromosomal positions. CRISPR arrays and *cas* genes are adaptive bacterial immunity systems, particularly regarding anti-phage mechanisms. Evaluating CRISPR elements contributes to identifying robust starter strains and facilitates stable fermentation of industrial-scale soy sauce production. In the future research, we will develop a methodology for rapid detection and classification of CRISPR elements by using PCR-RFLP.

## Data Availability Statement

The datasets presented in this study can be found in online repositories. The names of the repository/repositories and accession number(s) can be found below: DDBJ BioProject http://trace.ddbj.nig.ac.jp/BPSearch/bioproject?acc=PRJDB9642.

## Author Contributions

MM and AO conceived and designed the study, and performed the experimental procedures. MM analyzed all the data. MM, TW, and AO wrote the original draft of the manuscript. TW, JW, and MT supervised the experimental work, and reviewed and edited the manuscript. All authors approved the final manuscript.

## Conflict of Interest

TW and JW were employed by the company Yamasa Corporation. The remaining authors declare that the research was conducted in the absence of any commercial or financial relationships that could be construed as a potential conflict of interest.

## Footnotes

1 https://www.bioinformatics.babraham.ac.uk/projects/trim_galore/
